# A257 ASSOCIATION OF DIETITIAN CONSULTATION WITH INFECTION RATES IN PATIENTS WITH SEVERE ALCOHOLIC HEPATITIS

**DOI:** 10.1093/jcag/gwac036.257

**Published:** 2023-03-07

**Authors:** M T K Yau, C Tsien, B Bielawska

**Affiliations:** 1 Faculty of Medicine, University of Ottawa, Ottawa; 2 Amjera Transplant Centre, Toronto General Hospital, Toronto; 3 Division of Gastroenterology, Department of Medicine, The Ottawa Hospital, Ottawa, Canada

## Abstract

**Background:**

Patients with alcoholic hepatitis have significant morbidity and mortality, frequently infection related. They are often malnourished from increased nutritional demands. Studies show conflicting results on whether nutritional therapy reduces infections.

**Purpose:**

To examine if dietitian consultation is associated with lower infection rates in patients with alcoholic hepatitis.

**Method:**

Retrospective chart review of adults aged 18-75 admitted to The Ottawa Hospital for severe alcoholic hepatitis between January 1, 2016 and December 31, 2018. Patients with co-existing liver diseases were excluded. Descriptive statistics compared those with and without dietitian consultation during admission. The primary exposure was dietitian consultation over 48 hours before infection. The primary outcome was infection over 48 hours after admission.

**Result(s):**

64 patients were assessed (mean age 52.3+/-10.9 years; mean Maddrey Discriminant Function 53.6+/-33.2). Those with dietitian consultation (N=17, 26.6%) had greater Charlson (p=0.016) and Model for End Stage Liver Disease (p=0.048) scores, with no difference in steroid use (p=0.60). Among 53 patients without infection on admission, 10 had infection in hospital, of whom 4 had dietitian consultation before infection. Dietitian consultation was associated with increased infection risk (OR 6.5, 95%CI=1.3-33.2).

**Conclusion(s):**

Only the sickest patients received dietitian consultation, which likely explains the observed increased infection risk. Given high malnutrition rates in this population, our results suggest a significant care gap.

**Please acknowledge all funding agencies by checking the applicable boxes below:**

None

**Disclosure of Interest:**

None Declared

